# Experiences of Sexual and Intimate Partner Violence Among Women International Students in Australia

**DOI:** 10.1177/10778012251323267

**Published:** 2025-03-12

**Authors:** Laura Tarzia, Carolina Navarro Medel, Elizabeth McLindon, Paulina Ezer, Helen Forbes-Mewett, Ly Thi Tran, Adele Murdolo, Kelsey Hegarty

**Affiliations:** 1 Department of General Practice and Primary Care, 2281 The University of Melbourne, Carlton, VIC, Australia; 2 2282 The Royal Women's Hospital, Parkville, VIC, Australia; 3 School of Social Sciences, 2541 Monash University, Clayton, VIC, Australia; 4 School of Education and Research for Educational Impact (REDI) Centre, 2104 Deakin University, Burwood, VIC, Australia; 5 Multicultural Centre for Women's Health, Collingwood, VIC, Australia

**Keywords:** sexual violence, intimate partner violence, international students, Australia, women

## Abstract

Sexual violence (SV) and intimate partner violence (IPV) in tertiary institutions have received increased attention in Australia and globally, however, international students have been largely neglected in academic and policy discourse. Little is known about the nature and frequency of SV and IPV experienced by these students, nor what sociodemographic factors are associated with victimization. This article reports on a national cross-sectional survey of 1,491 women international students in Australia. Findings suggest that both SV and IPV are common among women international students and primarily perpetrated by men. Social support, housing stress, and financial insecurity were associated with an increased likelihood of victimization, highlighting critical areas for improvement in government policies and tertiary institutions.

## Introduction

Sexual violence (SV) and intimate partner violence (IPV) are major, overlapping social issues in Australia (Commonwealth of Australia, 2022). Primarily perpetrated by men against women, current national data suggests that around one in five women has experienced SV, and one in four has experienced IPV since the age of 15 (Australian Bureau of Statistics, 2021–22). SV is broadly defined as any sexual act carried out against a person's will, either by a known perpetrator or a stranger (World Health Organization, 2013). It includes sex obtained through coercion, blackmail, or threats, sex while a person is asleep or incapacitated by alcohol or substances, as well as unwanted touching or kissing (Borumandnia et al., 2020; Dartnall & Jewkes, 2013). IPV is defined as physical, psychological, financial, or sexual abuse perpetrated by an intimate partner or ex-partner, typically characterized by a pattern of fear and control (World Health Organization, 2013). Both SV and IPV are associated with serious, long-term physical and mental health outcomes (World Health Organization, 2013), the consequences of which cost the Australian economy approximately $21.7 billion each year (Commonwealth of Australia, 2022).

Global evidence suggests that women attending tertiary education institutions may be at particularly high risk of both SV and IPV (Fedina et al., 2018; Pengpid & Peltzer, 2020; Rodriguez et al., 2022; Rosenberg et al., 2019; Sanci et al., 2022; Steele et al., 2023). A recent systematic review and meta-analysis of global evidence by Steele et al. (2023), for example, suggested that 17.5% of women in higher education may have experienced sexual assault, with forced touching the most reported behavior. Less data is available for IPV, but studies have reported prevalence rates of between 1% and 31% for physical violence and between 10% and 71% for psychological abuse (Cho et al., 2020; Scherer et al., 2016). Tertiary education can be a period of transition, experimentation, and independence for many students (Sanci et al., 2022). Compounding this, the absence of supervision, a culture of regular social and leisure activities with high levels of alcohol and/or substance misuse (Sanci et al., 2022), and persistent inequities including financial stress and food insecurity (Arkoudis et al., 2018), can intersect with broader patterns of gender inequality (Rosa et al., 2021) to create violence-supportive environments (Steele et al., 2022).

Although there is limited prevalence data in the Australian tertiary education context—particularly for IPV—existing data does suggest that violence is a serious issue in need of urgent action (Heywood et al., 2022; Sanci et al., 2022; Zark et al., 2022). A national survey of 43,819 university students in 2021, for example, found that 16% had experienced sexual harassment and 4.5% had been sexually assaulted since starting university, with women and nonbinary students and younger students aged 18–21 at greatest risk (Heywood et al., 2022). Similarly, a study of over 14,000 university students (Sanci et al., 2022) found that around 7% had experienced lifetime forced sex or attempted forced sex, and nearly a quarter had experienced unwanted sexual contact. A further 22% reported ever experiencing fear of an intimate partner (Sanci et al., 2022).

Although the broader issues of SV and IPV in the tertiary education setting have received increased research and policy attention worldwide, far less is known about the SV and IPV experiences of international students (Bonistall Postel, 2020). A relatively small body of quantitative research has reported mixed prevalence rates among this cohort (Chaliawala et al., 2024; Fethi et al., 2023; Ortensi & Farina, 2020; Scholl et al., 2019). For example, a recent study by Chaliawala et al. (2024) found that 6.7% of a sample of 13,242 international students in the United States had experienced nonpartner SV, with female students at greater risk of victimization. Another study by Scholl et al. (2019) reported that 5.5% of a sample of 112 international students at a Midwestern college in the United States had experienced SV in the preceding 12 months, and 19.6% had experienced lifetime physical IPV. On the other hand, Fethi et al. (2023) found that, of a sample of 764 international students in Canada (67% female), 30% had experienced sexual harassment, 17% unwanted sexual contact, and 2% sexual coercion in the previous 12 months. These varying prevalence rates are likely to be due to differences in how SV and IPV are measured and how international student status is defined (e.g., visa status vs. being “foreign-born” or “visible minority”).

In Australia, robust quantitative data exploring the nature, context, and prevalence of SV/IPV among international students is almost entirely absent. Only three studies (Heywood et al., 2022; Sanci et al., 2022; Zark et al., 2022) have included international students as a subset of a broader university student cohort. These studies have addressed SV in a limited way, and only one study (Zark et al., 2022) focused on IPV. This lack of data is highly problematic given the structural issues reported by women international students (Forbes-Mewett & McCulloch, 2015; Tran et al., 2024) and other migrant groups (Vaughan et al., 2016) in qualitative studies, which may increase their vulnerability to violence victimization. For example, isolation from family and social support networks, visa issues and temporary residency status, language and cultural barriers, ineligibility for services, and a lack of familiarity with the service landscape are suggested as potential risk factors (Forbes-Mewett & McCulloch, 2016; Tran et al., 2024). A further gap is that research undertaken in Australia focuses exclusively on the context of higher education (university). Yet, large numbers of students (particularly international students) are enrolled in other types of courses such as vocational education and training (VET; Australian Government, 2024).

Taken together, these gaps suggest that existing research may not be accurately capturing a full picture of SV or IPV against international students in Australia, nor shedding light on the complex intersectional factors that may characterize their experiences.

In this article, we present findings from a large, national survey of women international students in Australia. It addresses the lack of robust data on SV and IPV in this cohort, guided by the following aims:

1. To identify the types of SV and IPV behaviors experienced by women international students.
2. To understand the relationship between victim/survivor and perpetrator for SV of any type and for forced or coerced sex in particular.
3. To explore factors associated with greater odds of experiencing SV or IPV among women international students.

To date, few studies have explored these critical questions, which are central to future research, policy, and practice in the international education and international student support areas.

### The Context of International Education in Australia

Australia is one of the most popular study destinations for international students. In 2023, over 589,000 international students were enrolled in Australian courses (Australian Government, 2024). China, India, and Nepal make up the vast majority of enrollments, with Colombia, the Philippines, Thailand, Brazil, and Vietnam also providing large numbers of students (Australian Government, 2024). Higher education is the most popular educational pathway representing 50% of enrollments, although there is also a large proportion of students (35%) enrolled in VET courses (Australian Government, 2024), a popular choice for those seeking to learn a trade or who want a more industry-focused qualification. VET courses can be delivered by public institutes (e.g., Technical and Further Education [TAFE]) or private providers (Private Registered Training Organizations [RTO]), and typically offer a certificate or diploma upon completion. Smaller proportions of students enroll in schools and English Language Intensive Courses for Overseas Students (ELICOS), which are a pathway into further tertiary studies (Australian Government, 2024). To date, research on SV and IPV in Australia has only been undertaken with students attending universities.

Unlike the United States, where the bulk of research into SV and IPV has taken place, students in Australia typically do not reside in college dormitories and generally attend tertiary education institutions close to home. Although residential colleges do exist, most students live at home with family members, rent out private residential dwellings together with other students ('share-housing”), or choose other privately operated residential accommodation options (e.g., purpose-built student apartments; Morris et al., 2023). For example, in the study by Sanci et al. (2022) mentioned above, over 85% of international students were living in a rented flat, apartment or house, while only 7% were staying in on-campus accommodation. A major contributor to this trend is the relative scarcity and unaffordability of college accommodation in Australia (Marginson et al., 2010; Wilson et al., 2023). Studies suggest that many international students would prefer to live on campus but are unable to afford to do so (Marginson et al., 2010).

International students’ living situations can influence their broader student experience (Llano-Suarez et al., 2023). The absence of close peer contact, for example, potentially reduces opportunities for social connection (Wilson et al., 2023) and a lack of regulation of the private rental market can render students vulnerable to exploitation (Marginson et al., 2010; Morris et al., 2023).

## Method

The study involved a cross-sectional anonymous online survey of women international tertiary students in Australia. Recognizing that, for some students, answering sensitive questions may be easier in their first language, the survey was offered in five languages other than English—Mandarin, Nepalese, Portuguese, Vietnamese, and Hindi. These languages reflected the main groups of international students in Australia at the time of the survey, although students from any background were able to participate. Ethics approval for this study was obtained by the Human Ethics Research Committee at The University of Melbourne (HREC #2057418.2).

### Eligibility

Participants were eligible for the study if they identified as a woman, were aged 16 years or older, and were an international tertiary student in Australia (i.e., someone from overseas currently enrolled in an Australian university or VET course, or in an ELICOS). Participants could be on a student visa, bridging visa, or partner/secondary visa (recognizing that people may enroll in tertiary education while in Australia, without this being the primary reason for their travel), but needed to be located on-shore (this was particularly important during the COVID-19 pandemic during which Australia prevented international students from entering the country). School students and students from New Zealand were not eligible. Further requirements included having the ability to read and write in at least one of the languages in which the survey was offered and having access to a device with an internet connection.

For inclusion in the analysis for this article, students needed to have answered “yes” or “no” for one or more questions on SV.

### Recruitment

Participants were recruited nationally using a range of strategies. These included:

- *Social media*: We used a variety of paid and unpaid advertisements on Facebook, Twitter, Instagram, Snapchat, WeChat, Weibo, and YouTube. Given the high incidence of fraud in many online surveys, we wanted to ensure that we were targeting our chosen demographic as much as possible rather than accessing a broader audience. Consequently, we focused on social media channels that were either specific to international students or to migrant communities. We also placed paid ads on many of the social media platforms.
- *Student newsletters and groups*: We placed advertisements on our institutional student noticeboard and liaised with the international student association on campus. We also reached out to equivalent associations at other tertiary education institutions.
- *Paid consultants and professional media organizations*: We hired a Chinese research assistant to support recruitment using WeChat. We also paid a professional media marketing company which specialized in advertising to the Chinese community, to place our ads on Chinese social media platforms, Weibo and Little Red Book.
- *Community partners*: We built up relationships with key community partners including international student organizations, multicultural services, legal services, and violence services. Partners promoted our study advertisements via their networks and on social media.
- *Key stakeholders*: By far the most successful strategy was the development of relationships with key stakeholders across the tertiary sector. We spent time identifying key contacts within as many tertiary education providers as possible, reaching out to these individuals with a formal letter of introduction to the study. Some universities were reluctant to engage with the project, given the recent political climate in Australia regarding sexual assault in the higher education sector. On the other hand, for other universities, involvement with the study provided an opportunity to strengthen their responses and gauge the effectiveness of their prevention activities. Several supportive institutions provided endorsement at the highest level and the researchers were able to work cooperatively with their Safe Communities team (or equivalent) to distribute the study information to international students. We also engaged with stakeholders such as overseas insurance providers (some of whom distributed our study materials to their members via their regular newsletter) and student accommodation providers.

Our study advertisements were designed in consultation with international students and project stakeholders with cultural inclusiveness and sensitivity in mind. We used images of women from diverse cultural backgrounds and emphasized the opportunity to win prizes. The wording for the advertisements read: “Are you an international student woman aged 16+? Please fill out an anonymous survey about health, well-being, consent, and relationships for a university study.” The use of broader language around “health, well-being, consent, and relationships” was deliberate, since many women do not label their experiences as “sexual violence” or “intimate partner violence” (Harned, 2005). We also used the terms “unwanted sexual contact” and “unhealthy relationships” throughout the survey and in study materials.

Potential participants followed a link from the recruitment material which directed them to the survey welcome page that contained information about the study. After reading this information and confirming consent, participants were asked: “Do you identify as a woman? Are you an international tertiary student? Are you 16 years of age or older?” We also included an easy mathematical question to ensure that bots were unable to progress. Those who met the initial criteria were invited to complete the survey. Those who did not meet the eligibility criteria were thanked and referred to the resource page for general information.

On completion of the survey, students were offered the option to enter a draw to win gift vouchers or an Apple iPad. Any contact information provided as part of the competition prize draw was collected using a separate Qualtrics survey, with the answers unable to be linked back to the main dataset.

### Survey Design and Measures

The survey was designed using the Qualtrics platform. It was composed of six main sections: About You; Your Health and Well-being; Your Relationships; Life Events; Getting Help; and More About You. We collected a range of sociodemographic data including age, visa status, country of birth, main language spoken, location (state), length of time in Australia, relationship status, and study level. We also asked a range of health and well-being questions; however, these analyses will be reported separately. The main variables of interest are described below:

*SV.* We defined SV as any unwanted sexual experience, with or without physical contact. To capture a wide range of behaviors, while still being mindful of potential cultural sensitivities, we developed our own instrument in consultation with international students. We asked them whether they had experienced any of 18 different types of SV since arriving in Australia, with yes/no answer options. These behaviors were organized across three categories: “Unwanted Sexual Contact,” “Coercion and Threats,” and “Sexual Assault and Rape.” Women were deemed to have experienced SV if they responded “yes” to one or more of these items. For each “yes” answer, women were additionally asked about the perpetrator's gender (male, female, nonbinary/transgender, not sure), the relationship with that person from a list of 10 options (e.g., a casual date or hookup, a partner or ex-partner, a lecturer/tutor/supervisor, a stranger), and whether the SV had occurred in the previous 12 months or longer.

*IPV.* For those participants who had been in an adult intimate relationship lasting longer than 1 month since the age of 16, we measured exposure to violence by an intimate partner using the Composite Abuse Scale (CAS; Hegarty et al., 2005). The CAS is a well-validated self-report measure of physically, sexually, and emotionally abusive behaviors in the previous 12 months. The scale asks about the frequency of the abusive behaviors using a 6-point scale and covers four categories of IPV: “Severe Combined Abuse” (severe physical, emotional, and/or sexual violence), “Physical and Emotional Abuse,” “Physical Abuse” alone (not in combination with any other category of abuse), and “Emotional Abuse and/or Harassment.” Students were deemed to have experienced IPV if they scored positively on any one of the four CAS categories. For each “yes” answer, students were asked whether it was a male partner or female partner who had perpetrated the behavior.

*Social support.* We used the six-item MOS Social Support Scale (Holden et al., 2014) to measure the strength of participants’ self-reported social supports. Respondents were asked to rate the availability of a supportive person in six situations on a scale of 1 to 5, with 1 indicating that someone was available “none of the time” and 5 indicating “all of the time.” For example, “If you needed it, how often is someone available to do something enjoyable with?” or “If you needed it, how often is someone available to share your most private worries and fears with?” Mean scores were categorized as “none or a little of the time” (≤2), “some of the time” (>2 and ≤3), “most of the time” (>3 and ≤4), and “all of the time” (>4 and ≤5). For risk factor analysis, we recategorized social support into a binary variable, with scores ≤2 indicating no or little support available and scores >2 indicating some support available.

*Housing and financial insecurity.* Housing insecurity was measured by a single yes/no question asking, “In the past 12 months have you ever been without a place to live?” Financial insecurity was measured by a single item asking, “How do you manage on your available income?” Respondents indicated their answer on a 5-point scale: 1 = *easily*, 2 = *not too badly*, 3 = *difficult some of the time*, 4 = *difficult all of the time*, 5 = *impossible*. Answers “difficult all of the time” or “impossible” were deemed to be indicators of financial insecurity.

### Data Collection

The survey, conducted between September 2021 and April 2023, was anonymous and voluntary. It took 20–30 min to complete, depending on whether the participant had experienced violence. Due to the sensitivity of the topic, the study was designed to ensure that any potential risks to participants were managed. This included presenting all students with a list of resources with contact details of relevant services and allowing them to skip questions. We also provided students with a phone number to contact a member of the research team should they require support during or after the survey. A protocol was developed to respond sensitively to any distress from participants who might contact the research team for support (although this did not occur). On completion of data collection, the complete dataset was exported from Qualtrics and imported into the statistical software program Stata (StataCorp, 2021) for data cleaning, scoring, and analysis.

### Data Analysis

Descriptive data, including means and standard deviations for continuous variables, and counts and percentages for binary variables, were generated for participant demographics and for each of the variables of interest. Experience of SV and IPV were coded as binary variables to enable comparisons between those who had experienced at least one incident of SV since arriving in Australia or scored positively on at least one of the CAS categories (coded as 1 on the SV and IPV variables), and those who had not (coded as 0 on the SV and IPV variables). Types of SV were coded as “Unwanted Sexual Contact,” “Coercion and Threats,” or “Sexual Assault and Rape” when participants reported at least one violent behavior for each category. Participants could score positively for more than one SV type. For some analyses, we recoded the SV variable into “Any SV” or “Forced or coerced sexual acts” (comprised of the “coercion and threats” and “sexual assault and rape” categories). The four types of IPV—Severe combined abuse, Physical abuse only, Physical and emotional abuse, and Emotional abuse only—were scored using standard 12-month cut-offs, as per the CAS guidelines (Hegarty et al., 2005). Perpetrator characteristics were coded when participants reported at least one violent behavior associated with each characteristic. Participants could indicate more than one perpetrator (e.g., reporting one SV behavior by a male perpetrator and another by a female perpetrator).

Logistic regression analysis was used to model the association between (binary) outcome and exposure variables through odds ratios (ORs; Kirkwood & Sterne, 2003). Specifically, the analysis focused on associations between experiences of SV and/or IPV and demographic variables selected *a priori*—low social support, housing or financial insecurity, and age (24 years or younger). The first three demographic variables were chosen because of their salience in the qualitative literature on the experiences of international students (Forbes-Mewett & McCulloch, 2016; Morris et al., 2023; Tran et al., 2024). Age was chosen because national-level data suggests that younger people aged 18–24 are at the greatest risk of experiencing both SV and IPV (Australian Bureau of Statistics, 2021–22). We hypothesized that students with lower levels of social support, higher levels of financial and housing insecurity, and those aged under 24 years would be more likely to have experienced SV and/or IPV than those who did not meet these criteria. ORs and 95% confidence intervals were used to assess the likely size of the association between SV and/or IPV and the four demographic variables.

## Results

### Participant Characteristics

There were 1,491 eligible responses to the survey. Most women in the sample (53.6%, 799) had experienced SV in Australia and/or IPV in the last 12 months; 239 of them (16.0%) experienced both forms of violence. Table 1 shows the demographics of the sample by experiences of SV and/or IPV. Nearly a third of all participants were born in China (28.8%, 461), 10.7% (172) in the Philippines, and 10.2% (163) in India. The majority had a first language other than English (82.0%, 1,323) and described themselves as heterosexual (76.3%, 1,153). Close to half were aged 25–34 years (41.3%, 664) and had been in Australia for more than 1 year (45.4%, 723).

**Table 1.** Demographic Characteristics of the Sample (*N* = 1,491).

	SV and/or IPV *n *= 799^a^		No violence *n *= 691^b^		International student population^c^
	*M (SD)*				%
Age (years)	25.5 (5.1)		25.51 (5.5)		
Region of birth (%)	*n*	*%*	*n*	*%*	
Western Pacific region^d^ (53.5%)	376	47.5	422	61.2	41
South-East Asia (20.5%)	171	21.6	135	19.6	34
Europe (8.6%)	85	10.7	44	6.4	4
Latin American and Caribbean (8.1%)	70	8.8	51	7.4	8
North America (4.4%)	48	6.1	17	2.5	2
Africa (2.6%)	24	3.0	15	2.2	1
Eastern Mediterranean region (1.6%)	18	2.3	6	0.9	3
First language other than English	171	78.6	599	86.7	−
State								
NSW (36.6%)	299	38.1	248	37.1	39.1
VIC (34.7%)	264	33.6	254	38.0	30.8
QLD (11.3)	110	14.3	59	8.8	13.5
WA (8.1%)	67	8.5	55	8.2	5.6
SA (4.1%)	26	3.3	36	5.4	6.0
ACT (1.3%)	8	1.0	11	1.6	2.6
NT (0.6%)	6	0.8	3	0.4	0.6
TAS (0.5%)	5	0.6	3	0.4	1.9
More than 1 year in Australia	441	55.2	234	33.9	−
Type of institution								
University (77%)	622	77.8	523	75.7	60
VET (18.6%)	142	17.8	136	19.7	35.1
ELICOS (2.4%)	18	2.3	18	2.6	3.2
Nonaward course (2.1%)	17	2.1	14	2.0	1.7
Course type^e^								
Undergraduate degree (42.5%)	264	42.4	223	46.6	59.2
Postgraduate coursework degree (46.9%)	287	46.1	250	47.8	46.4
Postgraduate research degree (10.6%)	71	11.4	50	9.6	6.3
Full time study^f^	714	89.4	617	89.3	−
In a relationship/married	439	54.9	300	43.4	−
Identify as heterosexual	605	75.8	527	76.6	−
Employed (full or part time)	378	53.2	260	43.6	−

*Note.* SV = sexual violence; IPV = intimate partner violence; CAS = Composite Abuse Scale.

Denominators vary due to missing responses; base = all survey participants who responded. Maximum missing data *n *= 89 (5.9%).

^a^ Participants who reported one or more SV behaviors or scored for one or more IPV category (CAS).

^b^ Participants who did not score for SV or IPV.

^c^ Estimates based on available international data for 2022 from the Department of Education and Erudera.com. This dataset includes students attending high school but excludes students on any visa type other than student visa. It is therefore only a rough guide.

^d^ The Western Pacific region incorporates 37 different countries including China, Vietnam, Mongolia, Laos, Cambodia, Malaysia, Japan, and Fiji.

^e^ Only participants who had attended university were asked this question (*n *= 1,225).

^f^ Although student visas are conditional upon a full-time course load, some students were in Australia on a partner visa.

Our study sample was broadly similar to the overall international student population in Australia for 2020–2022 in terms of distribution across states and territories, nationality (categorized by WHO region) and course type, although there were some minor differences. For example, we had a slightly higher proportion of participants from the Western Pacific Region (53.5% compared to 41%), and a smaller proportion of participants from South-East Asia (20.5% compared to 34%). We also recruited more students from the European, North American, and African regions, and fewer from the Eastern Mediterranean region. Due to challenges recruiting from private RTOs and TAFE institutions, we recruited proportionally fewer students enrolled in these courses (18.6% compared to 35.1%), with a higher proportion of university students compared to the broader population of international students.

### Experiences of SV in Australia

Of the 1,491 participants, 600 (40.2%) had experienced one or more incidents of SV since they moved to Australia (see Table 2). The most common behaviors reported overall were sexual comments or harassment (30.5%; 455), unwanted kissing, grabbing, touching, or rubbing (16.9%; 252), and repeatedly being pressured for sex after saying “No” (8.2%; 122). Close to one in five participants (17.9%; 264) had experienced forced or coerced sexual acts (including 11.4% [165] who had experienced sexual assault, rape, or attempted rape).

**Table 2.** Frequencies and Percentages of IPV and/or SV, Subtypes of Violence, and SV Behaviors.

	*N* = 1,491		
	*n*		*%*
Experienced SV since being in Australia *Type of SV*^a^	600		40.2
* Unwanted sexual contact*	548		36.8
* *Made sexual comments, stared at you or harassed you in a way that made you feel unsafe	455		30.5
* *Kissed, grabbed, touched or rubbed up against you in a sexual way when you did not want them to	252		16.9
* *Exposed their sexual body parts to you or masturbated in front of you when you did not want them to	95		6.4
* Coercion and threats*	188		12.8
* *Pressured you repeatedly for sex even after you said “No”	122		8.2
* *Made you show your sexual body parts to them when you did not want to	64		4.3
* *Made you look at or participate in sexual photos or videos when you did not want to or when you did not consent to it (e.g., secretly filming you)	41		2.8
* *Told you it was your duty or obligation to have sex with them	30		2.0
* *Made you have sex in exchange for something you needed (e.g., money, accommodation, good grades, a job)	25		1.7
* *Made you have sex by using their authority over you (e.g., husband, employer, or family member)	19		1.3
* *Made you have sex by threatening that something bad would happen if you said “No” (e.g., end the relationship, tell your family you were having sex, cancel your visa)	12		0.8
* *Made you have sex by threatening to physically hurt you or someone you care about if you said “No”	8		0.5
* Sexual assault and rape*	165		11.4
* *Touched your sexual body parts while you were asleep, drunk or drugged	97		6.7
* *Removed a condom during sex without your consent to increase their own pleasure	58		3.9
* *Had sex with you or put their fingers (or another object) inside you when you were asleep, drunk, or drugged	48		3.3
* *Choked or strangled you during sex without consent	28		1.9
* *Used physical force to touch your sexual body parts (e.g., held you down, hit you)	24		1.6
* *Tried to make you have sex using physical force (e.g., held you down, hit you) and did not succeed	17		1.2
* *Used physical force to make you have sex with them (e.g., held you down, hit you)	13		0.9
Total SV coercion, threats, sexual assault, and rape	264		17.9
	*N* = 979^b^		
Experienced IPV in last 12 months	438	44.7	
* Type of IPV in the last 12 months*			
* * Severe combined abuse	214	21.9	
* *Emotional abuse only	136	13.9	
* *Physical and emotional abuse	65	6.6	
* *Physical abuse only	23	2.4	
	*N* = 1,491		
Experienced SV since being in Australia and IPV in last 12 months	239	16.0	

*Note.* SV = sexual violence; IPV = intimate partner violence.

Denominators vary due to missing responses; base = all survey participants who responded. Maximum missing data *n *= 315 (18.6%) across IPV data only.

^a^Categories are not mutually exclusive, participants could belong to more than one category based on the behaviors they had experienced.

^b^Four hundred three participants were omitted as they had never been in a relationship (lasting ≥ 1 month).

Almost all international student SV survivors (97.2%, 583) indicated that the perpetrator was male (see Table 3). When looking at all types of SV together, the most common single perpetrator category was stranger (62.2%, 373), although close to the same proportion (59.7%, 358) were assaulted by a known person (a casual hookup, intimate partner, friend, or other acquaintance). When coerced or forced sexual acts were examined on their own, the proportion of violence perpetrated by strangers was lower (47.7%, 126). A large proportion of coerced or forced sex was perpetrated by casual dates or hookups (43.2%, 114), friends or acquaintances (37.1%, 98) and intimate partners (37.5%, 99).

**Table 3.** Perpetrator Characteristics by Experience of SV and IPV.

	Any SV (*N* = 600)		Forced or coerced sexual acts (*N* = 264)		IPV (*N* = 438)	
	*n*	*%*	*n*	*%*	*N*	*%*
Gender						
Male	583	97.2	259	98.1	412	94.1
Female	24	4.0	17	6.4	34	7.8
Nonbinary/transgender	5	0.8	5	1.9	−	−
Not sure	15	2.5	6	2.3	−	−
Relationship to victim/survivor						
Stranger	373	62.2	126	47.7	−	−
Friend or other acquaintance	169	28.2	98	37.1	−	−
A casual date or hook up	132	22.0	114	43.2	−	−
A partner or ex-partner	102	17.0	99	37.5	−	−
Employer or client	39	6.5	20	7.6	−	−
Lecturer/tutor/supervisor	6	1.0	3	1.1	−	−
A family member	2	0.3	2	0.8	−	−
Priest or religious leader	1	0.2	1	0.4	−	−

*Note.* SV = sexual violence; IPV = intimate partner violence.

Denominators vary due to missing responses; base = all survey participants who reported perpetrator characteristics associated with one or more SV/IPV behaviors. Values do not add to 100% because of the possibility of multiple violent episodes perpetrated by different people. Maximum missing data <5%.

### Twelve-Month Prevalence of IPV

As noted earlier, the CAS measures exposure to IPV over the preceding 12 months (Hegarty et al., 2005). Close to half (44.7%, 438) of the participants who had ever been in a relationship had experienced IPV in the previous 12 months (Table 2). The most common form of violence by an intimate partner during the previous 12 months was Severe Combined Abuse (21.9%, 214) followed by Emotional Abuse alone (13.9%, 136); Physical Abuse alone was the least reported form of IPV (2.4%, 23). The IPV behaviors that participants reported most included a partner telling them that they were not good enough (32.1%, 314), crazy (26.5%, 260), or stupid (21.6%, 212). A partner's use of a knife, gun, or other weapon was disclosed by 1.5% (15) of participants. Most international students (94.1%, 412) had experienced IPV by a male perpetrator (Table 3).

### Factors Associated With SV and IPV

We examined whether four variables—housing insecurity, financial insecurity, poor social support, and younger age—were associated with experiencing SV in Australia and/or IPV in the last 12 months. We found that women international students who had experienced housing insecurity were significantly more likely to have experienced SV (of any type) than participants not facing housing insecurity (18.9% vs. 11.0%) at increased odds of 1.9 (95% CI [1.4−2.6]). Financial insecurity was also associated with SV of any type (OR, 1.6, [1.2–2.4]), however, age and low social support were not. When we examined associations with forced or coerced sex in isolation, we found that women international students who reported having little or no social support had more than one and a half times the odds of reporting forced or coerced sex in the last 12 months (OR, 1.7, [1.1–2.6]) compared to women international students with stronger social support. Women international students experiencing financial insecurity (OR, 1.9, [1.3–2.9]) or housing insecurity (OR, 1.7, [1.2–2.5]) also had heightened odds of reporting forced or coerced sex.

Women international students who had little or no social support in Australia were more likely to report IPV in the last 12 months compared to those with greater social support, with increased odds of 2.3 ([1.3–4.2]). Compared to those unexposed to IPV in the last 12 months, IPV survivors were also more likely to report housing insecurity (OR, 1.8, [1.3–2.6]) and financial insecurity (OR, 1.8, [1.2–2.8]), but not younger age.

## Discussion

The findings of this study highlight that both SV and IPV are common among women international students. Over 40% of the participants had experienced at least one incident of SV since moving to Australia. One in five students reported experiences of coercion, threats, sexual assault, or rape. Almost all the participants had experienced SV perpetrated by a male perpetrator, primarily known individuals such as intimate partners, casual dates and hookups, and friends. Few international students reported SV perpetrated by an employer, despite workplace sexual harassment being flagged as a serious problem among broader cohorts of migrant and refugee women (Keel et al., 2023). This may be due to restrictive international student visa requirements around paid employment. Similarly, university lecturers/tutors were not commonly reported, in contrast to other studies (Fethi et al., 2023). Factors associated with experiencing SV (of any type) included housing insecurity and financial insecurity. For forced or coerced sex, there was an additional association with low social support. Almost half of the participants who had ever been in a relationship had experienced IPV in the previous 12 months. IPV was also associated with low social support, housing insecurity, and financial insecurity.

Our study directly and explicitly addresses the frequency, nature, and context of SV and IPV for women international students. With a few notable exceptions (Chaliawala et al., 2024; Fethi et al., 2023; Sanci et al., 2022; Zark et al., 2022), prior research on violence in the tertiary sector has either ignored international students or treated them as an afterthought; this has led to a poor understanding of their experiences (Bonistall Postel, 2020). Strengths of our study include a broad definition of SV encapsulating a range of behaviors across the spectrum, and a gold-standard, a validated measure of IPV that accounts for patterns of abusive behaviors in relationships. Moreover, we included international students from across the tertiary sector, not only those attending university, and included both postgraduate and undergraduate students. Although our sample was self-selecting, our study cohort was broadly representative of the overall international student population in Australia for 2020–2022. The availability of the survey in multiple languages is also a key strength that may have increased participation and comfort. At the same time, there are some important limitations. First, with a self-selecting sample, we cannot speak to the true prevalence of SV and IPV among women international students in Australia. The unfortunate reality was that access to student mailing lists from institutions was not possible due to privacy legislation. Second, the cross-sectional nature of our survey means that we could only examine associations with sociodemographic variables rather than determining causality. Third, a moderate level of missing data across the IPV items (16.5%) means that some experiences of violence may not have been captured. Fourth, our survey was open to international students who identified as women. As we did not ask students to report their sex or gender identity, we do not know the proportion of cisgender/transgender participants. Finally, it is worth acknowledging that our survey recruitment overlapped with the COVID-19 pandemic. Australian data does not suggest large increases in the frequency of SV or IPV during this period (AIHW, 2023), however, it is still possible that the timing of the survey impacted the findings.

Our findings speak to the worrying frequency of SV among women international students in Australia, underpinning the urgent need to direct greater attention and resourcing toward supporting these students. The rates of SV reported in our study are higher than those reported for international students in existing research in the Australian tertiary setting (Sanci et al., 2022), including the recent National Student Safety Survey (Heywood et al., 2022). They are also higher than many studies with similar behavior-based questionnaires undertaken with international students in different settings (Fethi et al., 2023; Ortensi & Farina, 2020). This may be due to the self-selecting nature of our sample, with students who had had an experience of SV being more motivated to complete the survey. However, given that we offered our survey in multiple languages and made efforts to ensure that the survey was framed in a way that was acceptable to international students (including extensive consultation on the wording of the SV items), it is also possible that participants felt more comfortable to respond than with prior studies.

A novel contribution of our study is that it sheds light on the specific types of SV experienced by women international students. The most common behaviors reported by participants were someone making sexual comments, staring, or behaving in ways that made them feel unsafe (30%), as well as unwanted groping, kissing, or touching (16%). Of particular concern were the 8.2% of students who reported repeated pressure for sex after saying “No” and the 10% of students who reported either being sexually penetrated or had their sexual body parts touched while asleep or under the influence of alcohol or drugs. Nearly 4% of students had been stealthed (nonconsensual condom removal). Contrary to what has been suggested in the extant literature (Forbes-Mewett & McCulloch, 2016), few students reported being blackmailed or threatened into sex (e.g., by withholding something they needed or weaponizing a position of authority). Rather, women international students were victimized in similar ways to domestic students—harassed by men in public spaces, or sexually assaulted by partners, friends, or dates they should have been able to trust. Choking or strangulation was reported only by 0.8% of students, offering a counter-narrative to recent scholarship that suggests this behavior is highly prevalent among young people in Australia (Sharman et al., 2024).

Nearly half of the students who had ever been in a relationship had experienced IPV in the past 12 months, which is also of serious concern. It is important to acknowledge that the 12-month timeframe indicated by the CAS is an imprecise proxy for time in Australia, and therefore it is possible that the true frequency of IPV within the cohort was even higher. Indeed, many of the students in our sample had been in Australia for longer than 1 year. The most common category of IPV was “severe combined abuse,” which is suggestive of a greater level of risk of harm (Hegarty et al., 2005). These findings highlight the need for the tertiary sector to address IPV alongside SV, including for international students. Given that international students are away from their usual support structures and face a number of cultural and systemic barriers to service access in the host country (Tran et al., 2024), tertiary education institutions need to consider how to support international students to remain safe while in Australia. As Zark et al. have argued (2022), standalone policies and resources could be developed for IPV—as they have been for SV—that are guided by trauma-informed principles and cultural responsiveness.

Housing insecurity (defined as not having somewhere to live in the past 12 months) and financial insecurity (defined as difficulty managing on current income) were the main sociodemographic factors associated with overall SV victimization among our sample. Although housing research on international students is lacking, studies have suggested that international students—particularly those from less wealthy backgrounds—can struggle to find secure, affordable, and safe housing in Australia (Morris et al., 2023). Morris et al., in a survey of over 7,000 international tertiary students, reported that a third found it difficult to meet weekly rent, and almost one in five had gone without basic necessities in order to pay rent (Morris et al., 2023). Similarly, studies suggest that international students can experience financial stress in the face of high tuition fees, ineligibility for social security supports, and employment conditions placed on student visas (Wilson et al., 2023). These pressures are likely to have been exacerbated during the COVID-19 pandemic, with one study finding a fivefold increase in international students’ odds of being unable to afford food (Russell et al., 2023). While it has been acknowledged that insecure housing and financial stress can impact students’ well-being and academic performance, the links between housing insecurity and the risk of experiencing SV have been underrecognized. Yet, as Morris et al. (2023) note, financial insecurity, housing affordability issues, and rental stress can drive students into unsafe housing arrangements and exploitative living conditions (Wilson et al., 2023). It is not difficult to imagine how this context could facilitate SV. On the other hand, since the directionality of the relationship is unclear, it could also be possible that experiences of SV could cause women international students to have to change accommodation frequently. Similarly, co-occurring financial abuse by a partner could cause financial insecurity (Mellar et al., 2024). Either way, it is an unfortunate reality that there are few strategies in place—either within tertiary education institutions or at the policy level—to ensure that international students are safely housed and financially secure; indeed, they are largely left to fend for themselves (Morris et al., 2023; Peterie et al., 2024). This is a key area for action to enhance student safety and potentially reduce the odds of SV victimization.

When forced or coerced sexual acts were examined separately, low or no social support was also found to be associated with greater odds of victimization. Poor social support was also associated with greater odds of experiencing IPV in the last 12 months. Again, qualitative research has shown that women international students are not eligible to use all support services in Australia, and can struggle to form support networks (Tran et al., 2024), and this leaves them isolated and uncertain of where to go when they experience violence. Students with low social support may not have anyone with whom to speak about issues in their relationship and be more likely to be dependent on the perpetrator and unable to leave. On the other hand, research with broader cohorts of women has consistently shown that perpetrators isolate women from their networks as a tactic of control in relationships (Stark, 2007). Consequently, it could equally be the case that experiencing IPV as an international student creates or exacerbates social isolation. It is critical that students be adequately linked in with multiple social supports upon arrival to Australia to potentially reduce the risk of victimization and to ensure that, if they do experience violence, they do not have to manage alone. Additionally, it is important that all support services that are generally available in the community are also available without restriction or cost to international students.

Wilson et al. (2023) have highlighted the relationship between low social support, financial stress, and housing insecurity, suggesting that this “three-domain framework” is central to understanding the precarity international students can experience. As they point out, international students (particularly those from low- or middle-income countries or impoverished families) are made vulnerable by the migration and visa process, exclusionary host government policies around employment and social security, and lack of the necessary social/emotional resources (i.e., strong social networks in the host country) to help them navigate study-related migration. They conclude that the combination of increased financial and housing stress and low social support can accelerate the risk of precarious events (Wilson et al., 2023). In line with their work, our findings highlight that each of the three domains adds to the odds of experiencing both forced or coerced sex and IPV, adding a gendered dimension to their analysis and making a novel contribution to the literature.

## Recommendations for Policy and Practice

Based on the findings of our research, we make the following recommendations:

We call on governments, policymakers, and tertiary education institutions to pay greater attention to the issues of SV and IPV for women international students. Our findings suggest that these issues are common, yet there is little acknowledgment of international student experiences in academic or policy discourse. This is highly problematic in light of qualitative evidence that suggests international students experience particular contextual and structural barriers that prevent help-seeking (Tran et al., 2024). Better coordination between governments, policymakers, tertiary education institutions, and community services is needed to tackle SV/IPV and other health and well-being risks faced by international students.

Our findings are indicative of high rates of IPV among international students. This is also the case for domestic students (Sanci et al., 2022). We suggest that national prevalence surveys also include IPV, and that tertiary education institutions develop policies to tackle this issue alongside SV. Given the high rates of SV perpetration by intimate partners, it is also vital that policies address the overlaps between the two forms of violence.

Tertiary education institutions need to take greater responsibility for the health and well-being of international students. Given the relationship between financial stress and violence victimization, there is a pressing need to identify and support international students experiencing financial stress. Although our findings cannot confirm the directionality of the relationship, in either case, there are benefits to identifying financially stressed students. We concur with Wilson et al. (2023) that tertiary education institutions could use an appropriate and ethically sensitive financial risk assessment tool to screen incoming international students. Similarly, identifying students experiencing housing insecurity could be an important component of effective responses to SV and IPV. Tertiary education institutions could do more to support students to find affordable, safe, and secure housing and avoid turning a blind eye to potentially exploitative housing conditions that may place students at risk of violence. Finally, campus services supporting students with financial or housing issues ought to sensitively screen any student who presents with high levels of financial stress or housing insecurity for co-occurring SV or IPV.

Ensuring that international students are linked-in with social networks may also help to reduce the risk of experiencing violence, or to ameliorate its impacts after it has occurred. Initiatives such as peer support groups, mentorship programs, or “buddy” systems where incoming international students arrive in the host country already connected to others could be trialed and evaluated. To reduce women international students’ isolation, vulnerability and dependence on perpetrators, it is important that social support, health, and welfare services are available free of charge and without restrictions to all international students.

Culturally sensitive, linguistically appropriate, and contextually specific education for international students around SV and IPV is recommended. Others have made similar recommendations in prior research (Bonar et al., 2020; Kennedy et al., 2024) and, indeed, in some countries there has been some headway in achieving this. In Australia, however, education around sexual consent and relationships tends to be delivered in a one-size-fits-all format (Fair Agenda & End Rape on Campus, 2023) and rarely offered in language. In making this recommendation, we also recognize the impact and importance of structural issues in increasing international students’ risk of victimization, however, it is vital that international students are made aware that SV/IPV can happen in Australia and are informed about their legal rights.

## Future Research

We call for more research to be undertaken globally to fill the knowledge gaps that remain around international students’ experiences of SV and IPV. Specifically, we suggest that more large-scale survey studies with international students are needed in order to better understand the magnitude and nature of the problem. These surveys should be offered in multiple languages, address both SV and IPV, and utilize behaviorally based measurement instruments that can be adapted for use with different cultural groups. We also need more data on the health and well-being impacts of SV/IPV for international students, as well as on protective factors. While a gendered analysis is important, it is also critical to explore the experiences of broader cohorts of international students, including men and gender-diverse individuals. Finally, testing and evaluation of interventions to prevent and respond to SV/IPV against international students is a key area for future research.

## Conclusion

This study makes an important contribution to the limited global literature on international students’ experiences of violence, highlighting the frequency of SV and IPV among a large national sample, identifying categories of perpetrators, and exploring sociodemographic factors associated with increased odds of victimization. Although our findings are primarily relevant to the Australian tertiary context, they also have salience for other similar countries with large international student numbers (e.g., USA, UK, Canada). Our study also has important implications for governments, policymakers, and tertiary education institutions in Australia (and elsewhere) seeking to strengthen support for international students.
